# Laryngeal Ganglioneuromatosis in a Child With Multiple Endocrine Neoplasia Type 2B (MEN2B): Case Report and Review of Literature

**DOI:** 10.7759/cureus.55406

**Published:** 2024-03-02

**Authors:** Yevgen Chornenkyy, Joseph Than, Saied Ghadersohi, Hector Melin-Aldana, Pauline Chou

**Affiliations:** 1 Pathology, Beth Israel Deaconess Medical Center, Harvard Medical School, Boston, USA; 2 Internal Medicine, NYC Health and Hospitals/South Brooklyn Health, Brooklyn, USA; 3 Pediatric Otolaryngology - Head & Neck Surgery, Ann and Robert H. Lurie Children's Hospital of Chicago, Chicago, USA; 4 Otolaryngology - Head and Neck Surgery, Feinberg School of Medicine, Northwestern University, Chicago, USA; 5 Pathology, Ann and Robert H. Lurie Children's Hospital of Chicago, Chicago, USA

**Keywords:** pediatrics, multiple endocrine neoplasia type 2b (men2b), ganglioneuroma, neuroma, vocal cord, larynx

## Abstract

Multiple Endocrine Neoplasia type 2B (MEN2B) is an autosomal dominant cancer syndrome caused by a mutation in rearranged during transfection (RET) proto-oncogene and includes medullary thyroid carcinoma, pheochromocytoma, gastrointestinal neuromas, and mucosal ganglioneuromas. Medullary thyroid carcinoma is the major cause of mortality in MEN2B syndrome. Medullary thyroid carcinoma can often appear during the first years of life. While mucosal neuromas in MEN2B are common, laryngeal neuromas are extremely rare. We present a third case of a pediatric patient with a laryngeal neuroma localized to the left true vocal cord and conduct a literature review of vocal cord neuromas in MEN2B patients.

## Introduction

Multiple endocrine neoplasia (MEN) type 2 is an autosomal dominant syndrome caused by a mutation in rearranged during transfection (RET) proto-oncogene and is associated with medullary thyroid carcinoma, pheochromocytoma, and hyperparathyroidism [1]. This syndrome is clinically classified into two phenotypes: MEN2A and MEN2B. MEN2B is associated with marfanoid habitus and multiple mucosal ganglioneuromas. While multiple mucosal neuromas on the lips, tongue, and buccal cavity are often observed, it is very rare for neuromas to appear in the larynx [1,2]. To our best knowledge, indexed literature between 1988 - 2020 contains only two other reports in the pediatric population [2,3]. Here we present the third pediatric case with ganglioneuroma on the vocal cord in a six-year-old male.

## Case presentation

**Figure 1:**
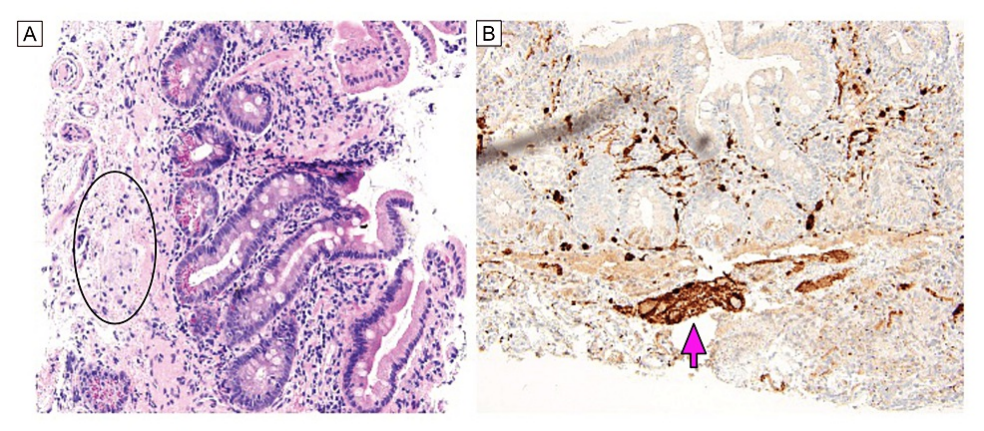
Gastrointestinal ganglioneuroma A: Small bowel biopsy with neuromatous-type tissue containing several ganglion cells adjacent to lamina propria (circled); B: Neuromatous-type tissue positive for S100 (arrow). (A, B – 200x).

**Figure 2:**
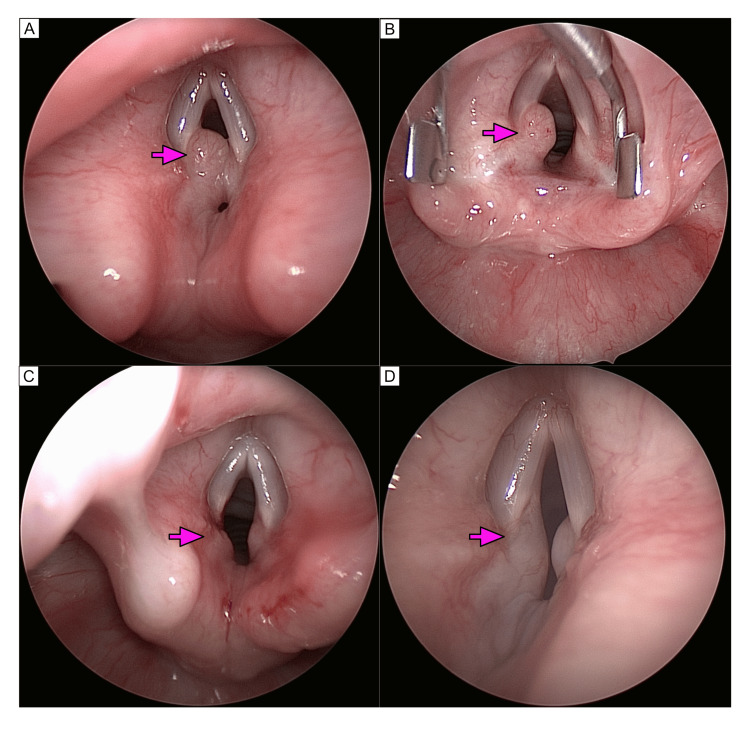
Endoscopic view of lesion before and after resection A: Initial endoscopic view of the vocal cords noting a moderately obstructive broad-based flesh-colored lesion emanating from the left posterior vocal cord (arrow). A smaller lesion is also seen from the right posterior vocal cord as well; B: Endoscopic view of the vocal cords with a vocal cord spreader in place noting a moderately obstructive broad-based flesh-colored lesion emanating from the left posterior vocal cord (arrow). C: Endoscopic view of the vocal cords after resection of the lesion from the left posterior vocal cord (arrow); D: Endoscopic view of the vocal cords 1 year and 6 months later. Noting a mild regrowth of the broad-based flesh-colored lesion emanating from the left posterior vocal cord (arrow). The lesion on the right posterior vocal cord remains stable in size.

**Figure 3:**
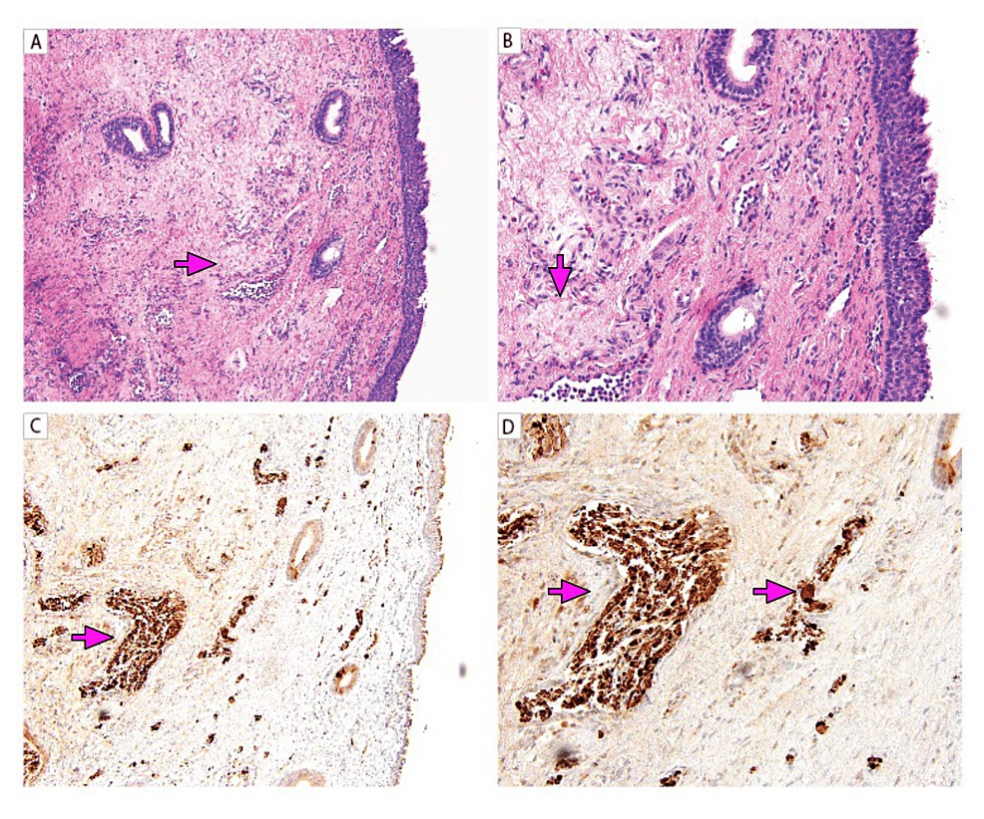
Vocal cord ganglioneuromas A, B: True vocal cord biopsy lined by ciliated stratified epithelium containing neuromatous-type tissue (arrow), nerve fragments, and ganglion cells; C, D: Neuronal-type tissue (arrow), nerve fragments, and ganglion cells (arrow) that are positive for S100. (A, C – 100X; B, D - 200X).

A six-year-old boy with a family history of MEN2B was diagnosed prenatally with RET M918T mutation. The patient underwent total thyroidectomy at six weeks revealing a 0.4 cm medullary microcarcinoma. Since birth, the patient’s clinical course was complicated by eosinophilic esophagitis, bowel ganglioneuromatosis (Figure 1), and dysmotility managed with colectomy and ileorectal anastomosis. At a follow-up visit with gastroenterology, incidentally, upon passing the scope, esophagogastroduodenoscopy (EGD) revealed a partially obstructive mass on the left true vocal cord (Figure 2). Otolaryngology service was consulted intraoperatively for assistance with airway management. After subtotal resection, pathology revealed a vocal cord ganglioneuroma (Figure 3). The patient is without symptoms of airway obstruction or dysphonia. Endoscopic view of the vocal cords at 1.5 years (Figure 3D), and ~3 years noted minimal regrowth.

Macroscopic findings

The glottis showed a 0.4 cm fleshy, tan, non-vascular left posterior true vocal fold lesion (TVC) with a broad base of attachment extending from the posterior third of the left TVC to the posterior midline glottis. The superior aspect of the underlying arytenoid with candidate nodule was left undisturbed, additionally, there was an opposing 0.15 cm right true vocal fold lesion.

Histological and immunohistochemical findings

The EGD revealed intestinal neuromatous-type tissue with several ganglion cells. The true vocal cord mass consisted of a polypoid piece of tissue lined by ciliated stratified epithelium. The stroma of the mass contains neuromatous-type tissue, nerve fragments, and ganglion cells, highlighted by an S100 stain.

## Discussion

Multiple endocrine neoplasia type 2 (MEN2) is an autosomal dominant disorder and contains three distinct cancer syndromes. In patients with familial medullary thyroid carcinoma (FMTC), only the thyroid is affected, while patients with MEN2A develop MTC, pheochromocytoma, and primary hyperparathyroidism. Patients with MEN2B are susceptible to developing MTC, pheochromocytoma, and ganglioneuromas of the digestive tract, mucosal neuromas, skeletal and eye abnormalities [3].

The underlying defect in patients with MEN2 disorders is a germline mutation in RET proto-oncogene on chromosome 10 [4]. While mutations at several codons are associated with MEN2A and FMTC, like A883F on exon 15, greater than 90% of MEN2B carry a germline mutation at M918T in exon 16 [4].

Manifestation of mucosal neuromas in MEN2B patients at diagnosis and follow-up occurs in 60% and 100% of patients before the age of six, respectively [5]. However, laryngeal neuromas are extremely rare. We present a third case of a pediatric patient with a laryngeal neuroma localized to true vocal cords and conduct a literature review.

Although mucosal neuromas have been widely observed on the lips, tongue, and buccal mucosa of patients with MEN2B, there has been limited documentation of laryngeal mucosal neuromas in the larynx [2]. The exact incidence of laryngeal mucosal neuromas is uncertain [2]. Between 1988 - 2020 seven cases of laryngeal neuromas in MEN2B patients have been reported with only four that are localized to the vocal cords (Table 1). Of the reviewed case reports, two patients (2/4) were >18 years old, and two (2/4) were <18 years old. The common presenting symptoms are dysphonia (2/4) [3,6] and hoarseness (1/4) [7-9]. The remaining patient (1/4) was asymptomatic and vocal cord neuromas were discovered incidentally [2]. After resection, the symptoms resolved in three patients (3/4) [3,7], while one pediatric patient (1/4) had a recurrence of neuroma but remained stable [2]. The vocal cord neuroma in our patient was discovered incidentally and after subtotal resection, the patient is without symptoms of airway obstruction at 18 months follow-up.

**Table 1:** Summary of published literature of MEN2B patients with laryngeal neuromas. N/S: positive, mutation not specified; N/A: not available; *: cases with a neuroma localized to the vocal cord; **: gender not specified; MEN2B - Multiple Endocrine Neoplasia type 2B

Author	Year	Summary	RET
Kudo et al. [1]	2014	A 20-year-old female presented with anterior left neck swelling and was found to have bilateral arytenoid neuromas.	N/S
*Ghosh et al. [2]	2007	An 11-year-old female with MEN2B who six years later was found to have incidental laryngeal mucosal and vocal cord nodules that recurred after resection and remained stable.	M918T
*Qualia et al. [3]	2007	A 3-month-old male initially presenting with gastrointestinal pseudo-obstruction treated with symptomatic management. At age 7 presence of oral neuromas initiated additional workup leading to a diagnosis of MEN2B. Status post total thyroidectomy patient developed dysphonia and a physical exam revealed left vocal cord neuroma. Dysphonia resolved after neoplasm resection.	N/S
*Soroa-Ruiz et al. [6]	2014	A 28-year-old female diagnosed with MEN2B at 17 years of age, with bilateral vocal cord neuromas on the exam for intermittent dysphonia. Neuromas did not recur after resection.	N/A
*Tolkemitt et al. [7]	2001	58-year-old patient** with increasing hoarseness for 3 months status post total thyroidectomy found to have right-sided nodules on the arytenoid cusps and the aryepiglottic folds. Nodules on vocal folds and arytenoid cartilage were excised. The patient’s voice function returned to almost normal 4 weeks after resection.	N/A
Lesourd et al. [8]	1988	A 28-year-old female with multiple mucosal neuromas present in the mouth, nasopharynx, and larynx was identified on a post-mortem examination after the patient passed metastatic medullary carcinoma.	N/A
McClurg et al. [9]	2015	A 30-year-old female with dysphagia, dysphonia, and cough. Physical examination demonstrated multiple laryngeal mucosal nodules throughout the glottis and supraglottis.	N/A

Imaging modalities aimed at reducing aerosol generation during the COVID-19 pandemic, while visualizing the vocal cords to detect laryngeal abnormalities, include laryngeal ultrasound, computerized tomography (CT), and magnetic resonance imaging (MRI) [10-11]. Mucosal laryngeal neuromas can potentially compromise the airway in patients with MEN2B. Although they are very rare, it should be remembered that neuromas can occur at this location in patients with MEN2B. Knowledge of this permits the management of this treatable condition.

## Conclusions

Multiple Endocrine Neoplasia type 2B (MEN2B) is an autosomal dominant cancer syndrome caused by a mutation in rearranged during transfection (RET) proto-oncogene. While mucosal neuromas in MEN2B are common, vocal cord neuromas are extremely rare. To our knowledge, this is the third reported pediatric case of vocal cord neuroma. Although rare, laryngeal mucosal neuroma can manifest as potentially airway-compromising lesions in patients with MEN2B, with resection being the management of choice in symptomatic cases.
